# Protective Effect of *Anoectochilus formosanus* Polysaccharide against Cyclophosphamide-Induced Immunosuppression in BALB/c Mice

**DOI:** 10.3390/foods12091910

**Published:** 2023-05-07

**Authors:** Anqi Xie, Hao Wan, Lei Feng, Boyun Yang, Yiqun Wan

**Affiliations:** 1 State Key Laboratory of Food Science and Technology, Nanchang University, Nanchang 330047, China; xieanqi2023@163.com (A.X.); wanhao424@ncu.edu.cn (H.W.); 2 School of Life Sciences, Nanchang University, Nanchang 330031, China; yangboyun@163.com; 3 Jiangxi Province Key Laboratory of Modern Analytical Science, Nanchang University, Nanchang 330031, China

**Keywords:** *Anoectochilus formosanus*, polysaccharide, immunosuppression, bioactivities

## Abstract

In this study, *Anoectochilus formosanus* polysaccharide (AFP) was acquired a via water extraction and alcohol precipitation method. The immunoregulatory activity of AFP was first evaluated on cyclophosphamide (Cy)-treated mice. Galacturonic acid, glucose and galactose were confirmed to be the main components of AFP. AFP demonstrated the ability to stimulate the production of TNF-α and IL-6 in RAW 264.7 macrophages. Not surprisingly, the activation of the NF-κB signaling pathway by AFP was validated via Western blot analysis. Furthermore, AFP could alleviate Cy-induced immunosuppression, and significantly enhance the immunity of mice via increasing the thymus index and body weight, stimulating the production of cytokines (IgA, IgG, SIgA, IL-2, IL-6 and IFN-γ). The improvement in the intestinal morphology of immunosuppressed mice showed that AFP could alleviate Cy-induced immune toxicity. These results have raised the possibility that AFP may act as a natural immunomodulator. Overall, the study of AFP was innovative and of great significance for AFP’s further application and utilization.

## 1. Introduction

Over the past decade, the burden of cancer in the world has been increasing continuously [1]. Cyclophosphamide (Cy) is an effective anticancer drug commonly used in the clinic [2]. However, the side effects caused by Cy should not be underestimated. The most common clinical symptom is that Cy has a strong immunosuppressive effect on human body [3]. Intestinal mucosal injury is one of the main manifestations of immunosuppression. Cy not only inhibits the secretion of immune cytokines in mice intestinal mucosa, resulting in immune dysfunction and intestinal injury, but also destroys the intestinal mucosal barrier and affects the integrity of the epithelium and neighboring cell–cell junctions, thereby impacting the absorption of food in the intestine [4,5,6,7].

Faced with these constraints, people have focused on polysaccharides derived from natural sources. Studies have proven that polysaccharides have extensive biological functions, such as promoting and protecting intestinal health [8], supporting normal bowel function, maintaining the regular state of blood glucose and lipids [9], and enhancing immunity [10]. Moreover, polysaccharides can be used as an anti-tumor and anti-inflammatory drug [11,12]. As an immunomodulator, polysaccharides have no significant side effects on the body [3]. It has been reported that the polysaccharides extracted from yellow pear residue markedly improve the immune function of mice [13].

The genus *Anoectochilus* (Orchidaceae) is a very precious perennial herb with 40 species worldwide, mainly distributed in China, Japan, India, Nepal, Sri Lanka, and other Asian countries. It is regarded as a traditional Chinese medicine and as beneficial to cancer, hypertension, diabetes mellitus, consumption, and nephritis [14,15,16]. At present, the major research species in China are produced in Fujian, Zhejiang, Jiangxi, and Taiwan [17]. *Anoectochilus formosanus* (AF) is one variety in the *Anoectochilus* family. According to reports, the water extract of AF has been shown to act not only as a protective agent in the liver, but also as an important immunomodulator [18]. In addition, studies have pointed out that the methanol extract of AF shows potential applicative value in the immunochemical prevention/treatment of cancer via the lowering of blood glucose, the scavenging of ROS, and the inhibition of PD-L1 [19]. Polysaccharides extracted from different parts of AF have shown different antidiabetic activity in vivo due to the different *M_w_* and monosaccharide compositions [20]. However, the reports on *Anoectochilus formosanus* polysaccharide (AFP) are relatively superficial. Little is known about the protective effects of AFP against immunosuppression. Therefore, exploring the bioactivity of AFP is of great significance in order to elucidate its function and utilization.

Based on these questions, AFPs were isolated from AF and their chemical properties were further investigated. The protective effect of AFP against the immunosuppression of mice induced by Cy was also invested. Therefore, our study was conducted in order to explore the immunomodulatory activities of AFP and provide a theoretical basis for developing AF.

## 2. Materials and Methods

### 2.1. Materials

*Anoectochilus formosanus* was obtained from Fujian province and verified by Prof. Boyun Yang from the Life Science Center of Nanchang University. It was then dried at 55 °C for 48 h before preparation. The monosaccharide standards used, D-Mannose, L-Arabinose, D-Ribose, etc., were purchased from Merck Co. (Darmstadt, Germany). Levamisole hydrochloride and cyclophosphamide were purchased from Aladdin Industrial Inc. (Shanghai, China). Cytokine (IgA, IgG, SIgA, IL-2, IL-6, IFN-γ, TNF-α) detecting ELISA kits were purchased from Biosharp biological technology Co., Ltd. (Shanghai, China). Lipopolysaccharide (LPS) and PBS buffer powder were purchased from Beijing Solarbio Science & Technology Co., Ltd. (Beijing, China). Commercially available analytical grade reagents were used in this study.

### 2.2. Extraction of Polysaccharides

Fresh plants were used as raw material and dried at 55 °C in the air-drying oven. After mechanical crushing, the coarse powder was passed through 80-mesh sieves to obtain a fine powder. Fat, pigment and small molecule were removed by soaking the powder in 95% ethanol for 10 h; this was repeated 4 times. After soaking, the powder was centrifuged and dried. The dried powder was extracted 4 times with distilled water at 80 °C with a material-to-liquid ratio of 1:20 (*w*/*v*) for 6 h. The supernatant was collected and the condensed solution was obtained by using a rotary evaporator under vacuum at 55 °C. Then, 85% ethanol was used to precipitate the polysaccharides with vigorous stirring; this was then stored at 4 °C overnight. The precipitate was collected after 10 min of centrifugation and washed three times with ethanol and acetone in succession. Finally, the precipitates were solubilized in distilled water and dialyzed (cut-off *M_w_* 3500 Da) for 48 h with distilled water, and then concentrated and lyophilized to obtain the polysaccharide named AFP [21].

### 2.3. Homogeneity and Molecular Weight Distribution of AFP

An Agilent 1260 Infinity system (Agilent Technologies, Amstelveen, The Netherlands) equipped with a refractive index detector (RID, G1362A) was used to measure the homogeneity and molecular weight of polysaccharide fractions via the method of high-performance gel permeation chromatography (HPGPC), according to a previous study [22,23].

### 2.4. Determination of Chemical Components

Glucose was treated as the standard in order to verify the neutral sugar content, using the phenol-sulphuric acids method [24]. The meta-hydroxydiphenyl method was performed according to a previous report, but with some modifications, to determine the uronic acid content in AFP. D-galacturonic acid was used as a standard [25]. In addition, protein content was determined using bovine serum albumin as a standard according to the Coomassie Brilliant Blue method [26]. Fat content was analyzed via the Soxhlet method [27].

### 2.5. Monosaccharide Composition Analysis

AFP monosaccharides were identified according to a published report but with some amendments [28], and the samples were analyzed on an Agilent 1260 Infinity system equipped with a UV detector (250 nm) and a Diamonsil C18 column (4.6 mm i.d. ×250 mm, 5 μm, Dikma, Foothill Ranch, CA, USA). The temperature of the column was maintained at 35 °C. The mobile phase was composed of acetonitrile and 0.1 mol/L PB (pH 6.8) in a ratio of 17:83 (*v*/*v*) at a flow rate of 1.0 mL/min. The injection volume was 20 μL, and the time of data collection was 50 min. Briefly, the AFP (5 mg) was hydrolyzed in 4 mL of 3 M TFA at 100 °C for 6 h in a sealed glass tube. After hydrolysis, the products were cooled to room temperature and dried with nitrogen. Diluting them with 1 mL of ultra-pure water, the hydrolysates were then obtained as derivatized, filtrated, loaded samples and monitored with the UV detector at an absorbance of 250 nm.

### 2.6. Fourier Transform Infrared Spectrum

A Thermo Nicolet 5700 infrared spectrophotometer (Thermo Electron, Madison, WI, USA) was used to characterize the organic functional groups of AFP using the KBr-pellets method, with the range of 400–4000 cm^−1^ [29].

### 2.7. Cell Culture

The murine macrophage RAW 264.7 cell was cultured in Dulbecco’s Modified Eagle’s medium (DMEM) medium containing 13% (*v*/*v*) fetal bovine serum (FBS). The cells were grown in a humidified incubator at 37 °C with an atmosphere comprising 5% CO_2_.

#### 2.7.1. Cell Viability

The Cell Counting Kit-8 (CCK-8) method was used to verify the cell viability. RAW 264.7 cells (2.5 × 10^5^ cells/well, 100 µL) were plated in 96-well plates for 24 h, followed by incubation with 100 µL of AFP (50, 100, 200, and 400 μg/mL) or lipopolysaccharides (LPS) (1 μg/mL) dissolved in DMEM medium for another 24 h. Subsequently, each well was incubated with 100 µL of diluted CCK-8 solution for another 2 h. Using a microplate reader, absorbance was measured at 450 nm. The experiment was carried out in triplicate.

#### 2.7.2. Influence of AFP on TNF-α and IL-6 Production of RAW 264.7

RAW 264.7 macrophage cells were plated into a 96-well plate with a density of approximately 2.5 × 10^5^ cells/mL and stimulated with AFP (100, 200, and 400 μg/mL) and LPS (1 μg/mL). TNF-α and IL-6 were detected in RAW 264.7 cells after 24 h of incubation with a commercial ELISA kit. The experiment was carried out in triplicate.

#### 2.7.3. Western Blot Analysis

RAW 264.7 cells were co-cultured with different concentrations of AFP (100, 200, and 400 μg/mL) and LPS (1 μg/mL) dissolved in DMEM medium for 24 h. The precipitates of the different treated cells were obtained using a centrifuge. The cell protein was prepared by adding 250 µL of Radio Immunoprecipitation Assay Lysis buffer (RIPA) lysate per 10^6^ cells. The denatured proteins were transferred by sodium dodecyl sulfate-polyacrylamide gel electrophoresis (SDS-PAGE) electrophoresis onto polyvinylidene fluoride (PVDF) (0.45 μm) membranes pre-activated with methanol and subsequently subjected to immunoreactivity. The PVDF membranes were treated successively using the primary antibody (anti-p-NF-κB p65, anti-NF-κB p65) and secondary antibody, followed by an elution with Tris Buffered Saline with Tween 20 (TBST) 3 times. Electrochemiluminescence (ELC) was used to visualize the protein band. Finally, the images were sorted and decolored to analyze the optical density values of the target bands.

### 2.8. Animals’ Experiments

#### 2.8.1. Animals and Treatment

SPF female BALB/c mice (20 ± 0.2 g, 6–8-week-old) were purchased from the Hunan Slac Jingda Laboratory Animal Co. Ltd. (Hunan, China), with the certificate number SYXK (Xiang) 2019-0004. All mice were maintained in an appropriate environment with free access to standard rodent chow and water at 22 ± 2 °C, with 60 ± 5% relative humidity, and a 12 h light/12 h dark cycle. The guidelines of Regulations for the Administration of Affairs Concerning Experimental Animals were strictly followed, and the study was approved by the State Council of the People’s Republic of China. After one week of acclimatization in the laboratory, all the mice were randomly divided into six groups, as follows: normal control group (denoted as NC group), model control group (denoted as MC group), AFP low-dose group (denoted as AFPL group), AFP medium-dose group (denoted as AFPM group), AFP high-dose group (denoted as AFPH group) and positive control group (denoted as PC group). Each group contained 12 mice, which were weighed every experiment day. The volume of intraperitoneal injection or gavage was one percent of the body weight of mice. For the first three consecutive days, the NC group received normal saline only as a normal control, while the other five groups received an intraperitoneal injection with Cy at a dose of 80 mg·kg^−1^·d^−1^ to construct a model of immune suppression [10]. The NC and MC groups received only normal saline intragastrically for the next seven days, while the AFPL, AFPM, and AFPH groups were given 50 mg·kg^−1^ BW, 100 mg·kg^−1^ BW, and 200 mg·kg^−1^ BW by gavage, respectively. Levamisole hydrochloride (LH) at 40 mg·kg^−1^ BW was administered by gavage to the PC group.

All mice were weighed and sacrificed 24 h after the last drug administration. The serum was obtained and the intact thymus was isolated via dissection. The small intestine was divided into segments according to the needs of different experiments. All anatomical tissues were frozen at −80 °C for subsequent analysis.

#### 2.8.2. Influence of AFP on Body Weight and Thymus Index

The weight of the mice was recorded every experimental day. After sacrificed, the intact thymus of the mice was harvested, rinsed with normal saline and blotted on filter paper before being weighed. Based on the reference formula, the thymus index was calculated as follows: thymus index = weight of thymus (mg)/weight of the body (g) [30].

#### 2.8.3. Preparation and Staining of Intestinal Section

At this stage, 4–6 cm jejunal tissue was cut and fixed with 10% neutral formalin for over 48 h. The tissues were dehydrated in a graded concentration of ethanol and embedded in paraffin wax afterwards. Samples were sliced into 4 μm thick paraffin sections for further analysis.

For hematoxylin-eosin staining (H&E) staining, paraffin sections were first dewaxed with xylene, absolute ethyl alcohol, and a gradient concentration of ethanol, respectively. Next, the sections were washed with distilled water. Secondly, the nucleus was stained with hematoxylin, and the cytoplasm was stained with Eosin. Thirdly, the sections were made transparent with xylene and then observed for histological changes. The H&E staining results were observed via an optical microscope at a 200-fold field of view. The complete villus and crypts should be present in each random magnified field of view.

For periodic acid schiff and alcian blue stain (AB-PAS) staining, after dewaxing and washing, the tissues were colored via immersion in 1% Alcian blue for 10–20 min, then running water was applied to rinse the tissue for 6 min. Following 10 min of oxidation with 0.5% periodic acid, the tissues were rinsed with running water for 6 min. Afterwards, the tissues were placed in the darkness and dipped in Schiff reagent for 15–30 min. Lastly, the samples were dehydrated, made transparent, fixed, and finally the AB-PAS staining results were observed using an optical microscope at a 200-fold field of view.

#### 2.8.4. Analysis of Serum Immunoglobulin A (IgA) and Immunoglobulin G (IgG) Secretion

The secretion of serum IgA and IgG was determined using ELISA Kits. The manufacturer’s instructions were strictly followed during all operating procedures.

#### 2.8.5. Analysis of Small Intestinal Cytokines Level

The production of small intestinal cytokines (secretory immunoglobulin A, SIgA; interleukin-2, IL-2; interleukin 6, IL-6; interferon γ, IFN-γ) was evaluated using ELISA Kits. The manufacturer’s instructions were strictly followed during all operating procedures.

### 2.9. Statistical Analysis

Experimental data were analyzed and expressed as mean ± standard deviation (SD) using SPSS22. IBM SPSS22 (SPSS Inc., Chicago, IL, USA) software was used to assess the statistical differences between groups via one-way analysis of variance (ANOVA) and the Tukey test. *p* < 0.05 indicated that the difference was statistically significant.

## 3. Results

### 3.1. Characterization and Identification of Polysaccharide from AF

**Figure 1:**
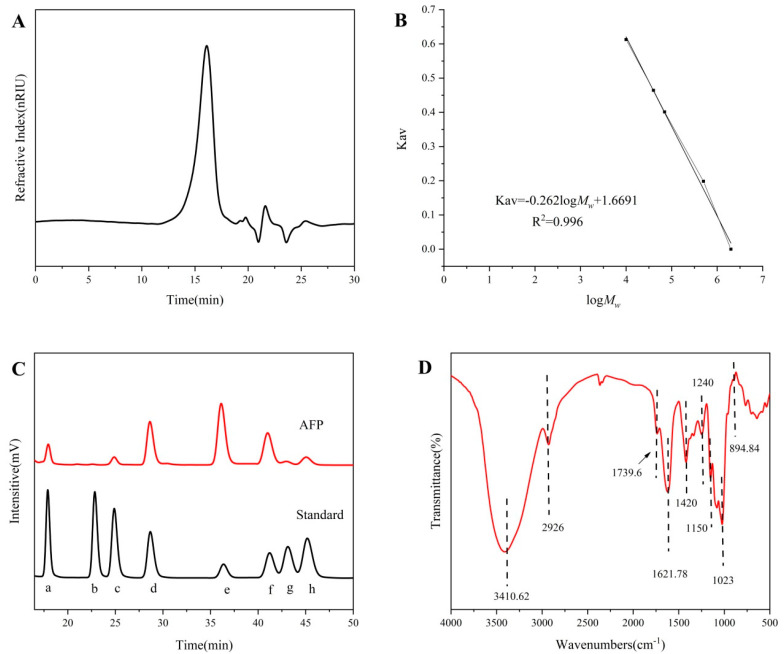
High-performance gel permeation chromatography profiles of AFP (**A**), and the HPGPC calibration curve of dextran standards and glucose (**B**). HPLC chromatogram profiles of monosaccharide composition of AFP (**C**). The monosaccharide standards (a) D-Mannose, (b) D-Ribose, (c) L-Rhamnose, (d) D-Galacturonic acid, © D-Glucose, (f) D-Galactose, (g) D-Xylose and (h) L-Arabinose, respectively, were used to qualitatively detect and analyze the samples. FT-IR spectra of AFP (**D**).

Hot water extraction and ethanol precipitation were used to extract the AFP. The yield rate of the AFP was designated as approximately 9.20% of the dry weight of raw material. AFP contained 46.70% neutral sugar, 2.98% protein, 25.07% uronic acid, and 0.23% fat. Single and symmetrical elution peaks were acquired using high-performance gel permeation chromatography (Figure 1A), indicating that AFP had relatively homogeneous components. Based on the standard regression equation of dextran and glucose (Figure 1B), AFP had a homogeneous *M_w_* distribution of 16.41 kDa.

### 3.2. Monosaccharide Composition Analysis

Figure 1C shows the monosaccharide composition of AFP. Based on the retention time of the monosaccharide standard, AFP was mainly composed of D-Mannose, D-Galacturonic acid, D-Glucose, and D-Galactose, accompanied by fewer amounts of L-Rhamnose, D-Xylose, and L-Arabinose. The mole ratios of D-Mannose, L-Rhamnose, D-Galacturonic acid, D-Glucose, D-Galactose, D-Xylose, and L-Arabinose were at 6.69%, 3.73%, 14.51%, 44.19%, 17.63%, 6.07% and 7.16%, respectively.

### 3.3. Fourier Transform Infrared Spectrum

The FT-IR spectra of AFP are shown in Figure 1D. The stretching vibration of O–H was the broad absorption peak at 3410.62 cm^−1^. Bands at 2926 cm^−1^ arose from C–H stretching vibrations, and the absorption between 1400~1200 cm^−1^ corresponded to the bending vibrations of C–H [31]. The absorption band of AFP centered at 1739.6 cm^−1^ was caused by an ester carbonyl C=O asymmetric stretching vibration, suggesting the existence of uronic acid [32]. The band at 1621.78 cm^−1^ was attributed to the -OH flexural vibrations of the polysaccharide. Furthermore, the absorption bands centered at 1150 cm^−1^, 1081 cm^−1^ and 1023 cm^−1^ were assigned to the stretching vibrations of the pyranose ring of the glucosyl residue [29]. In addition, the presence of β-type glycosidic linkages were suggested by the typical absorption at 894.84 cm^−1^ of AFP [33,34].

### 3.4. In Vitro Immunostimulatory Activities on Macrophages of AFP

**Figure 2:**
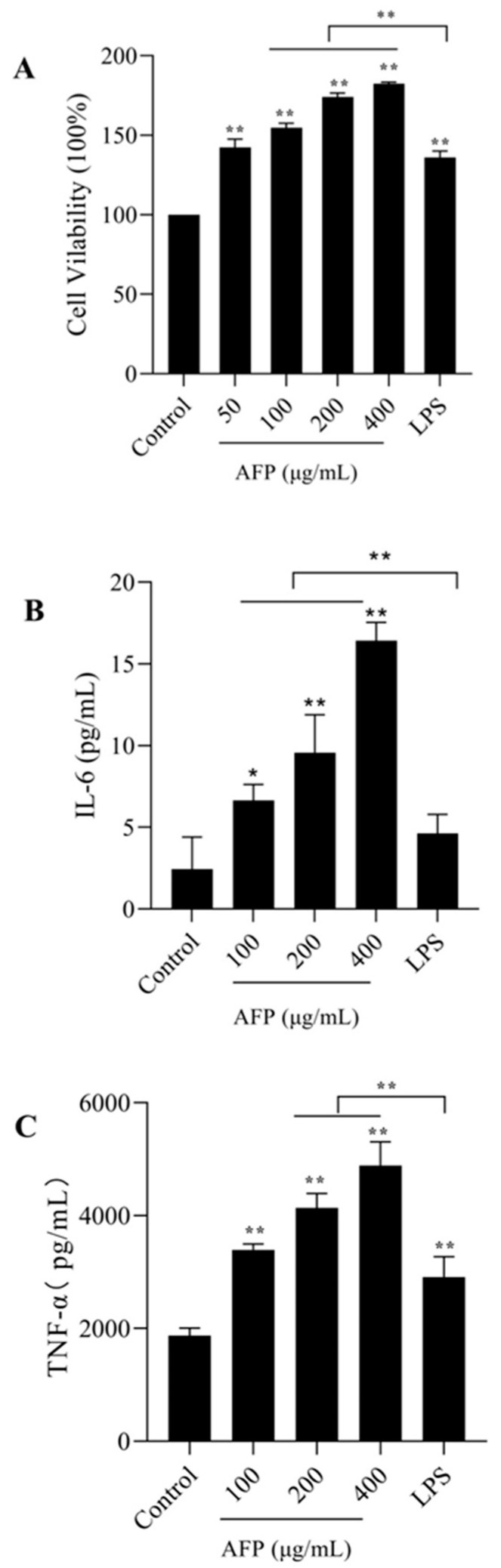
Effect of AFP on RAW 264.7 cells. (**A**) Cell proliferation rate of RAW 264.7 cells pretreated with AFP; (**B**) macrophages IL-6 secretion pretreated with AFP; (**C**) macrophages TNF-α secretion pretreated with AFP. Data are expressed as the mean ± SD (*n* = 3). * *p* < 0.05, ** *p* < 0.01 compared with the control group (0 μg/mL).

To explore the immunoregulatory effects of polysaccharides on macrophages, the ability of macrophages to proliferate in the presence of AFP in various concentrations was first measured. As shown in Figure 2A, the presence of AFP at various doses significantly enhanced the proliferation of macrophages in a dose-related pattern compared to the control group (*p* < 0.01). The highest proliferation ability was found at 400 μg/mL, which was up to 1.8 times the value of that of the control group. With respect to TNF-α, the expression increased with the increment in AFP from 100 to 400 μg/mL, but only notably upregulated TNF-α expression at 400 μg/mL (*p* < 0.05). Compared to the control group, AFP significantly increased IL-6 secretion in a dose-dependent manner (*p* < 0.05). The expression of IL-6 was elevated 8-fold by treatment with 400 μg/mL of AFP in comparison with the control group, and was evidently larger than those in the 100 and 200 μg/mL groups (*p* < 0.01).

### 3.5. Activation of the NF-κB Signaling Pathway by AFP

**Figure 3:**
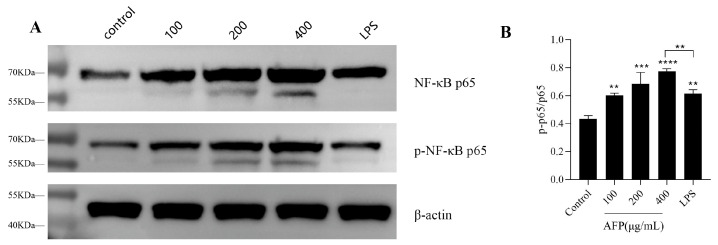
Effects of AFP on NF-κB signaling pathway in RAW 264.7 cells. (**A**) Western blot analysis of NF-κB induced by AFP in RAW264.7 cells. (**B**) Histogram represents the quantification of AFP-stimulated p-p65 in RAW264.7 cells. Data are expressed as the mean ± SD (*n* = 3). ** *p* < 0.01, *** *p* < 0.001, **** *p* < 0.0001 compared with the control group (0 μg/mL).

The immunomodulatory effect of AFP on RAW 264.7 was assessed by detecting the phosphorylation levels of NF-κB p65 in cells using Western blot analysis. The amount of p-NF-κB p65 was increased in accordance with the concentrations of AFP in a dose-dependent manner, as illustrated in Figure 3A. The phosphorylation level of NF-κB p65 was promoted by 1.41-fold after treatment with 1 μg/mL of LPS, relative to the control group (*p* < 0.01); this indicated that LPS could promote the phosphorylation of the NF-κB signaling pathway proteins markedly. Notably, the AFP group enhanced the amount of phosphorylation in the NF-κB p65 protein of the NF-κB pathway in a surprisingly good dose-dependent manner, in relation to the control group, and was even higher than that in the LPS group; this was responsible for the active inflammatory response. These results suggest that the NF-κB signaling pathway was a key signaling pathway for the immune activity of AFP in the macrophages.

### 3.6. Influence of AFP on Body Weight and Thymus Index

The variation in the body weight of the mice among the six groups was supervised every experimental day and the results are shown in Table 1. In comparison with the first day, the body weight of the Cy-treatment mice decreased dramatically on the fourth day (*p* < 0.05). During the remaining days of the experiment, the mice treated with AFP lost less body weight than those in the MC group. Namely, Cy-treated mice were associated with a risk of weight loss, whereas AFP could effectively reverse the weight loss caused by the Cy treatment.

**Figure 4:**
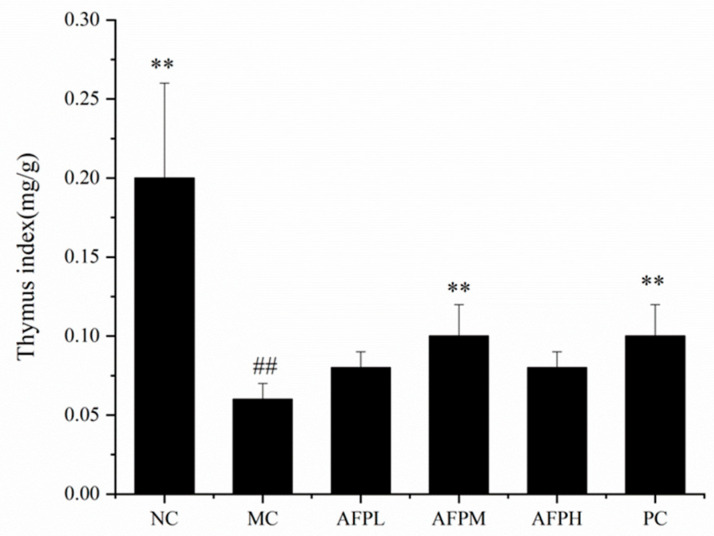
Effect of AFP on the thymus index in mice. Data are expressed as the mean ± SD, *n* = 10, ** *p* < 0.01 compared with MC; ^##^ *p* < 0.01 compared with NC.

The thymus index is exhibited in Figure 4. The MC group experienced a dramatic decline in the thymus index compared to the NC group (*p* < 0.01), indicating that Cy caused severe damage to the immune system of the mice. The thymus index of the AFP groups was raised significantly, relative to the MC group. The results demonstrated the immunoprotective effect of AFP in Cy-treated mice.

### 3.7. Effect of AFP on Intestine Tissue

**Figure 5:**
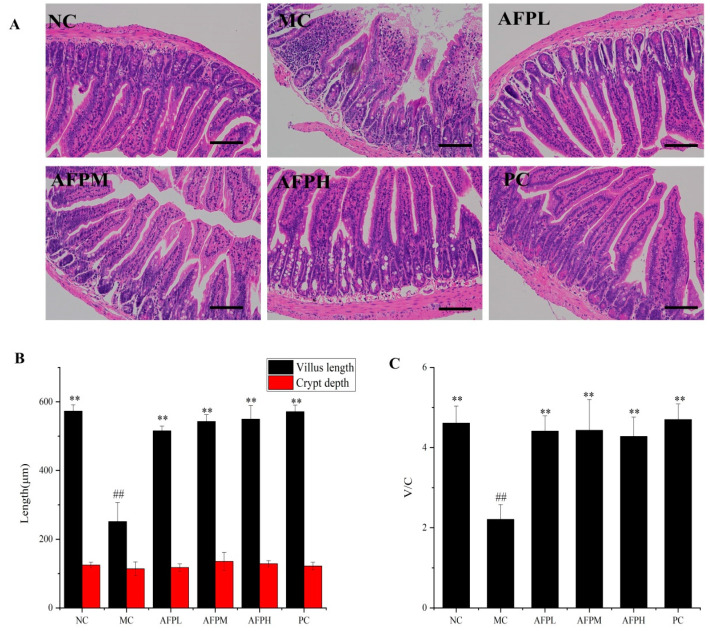
(**A**) Representative images of the small intestine tissue H&E staining sections (200×) among 6 mice, scale bar: 100 μm. (**B**) The villus length and crypt depth. (**C**) The ratio of villus length to crypt depth. Data are expressed as the mean ± SD (*n* = 6). ** *p* < 0.01 compared with MC; ^##^ *p* < 0.01 compared with NC.

To verify the impact of AFP on the intestinal morphology of Cy-immunosuppressed mice, hematoxylin and eosin (H&E) staining was conducted. In comparison with the NC group, relatively severe intestinal mucosa damage was revealed in the MC group; this was characterized by atrophic and edema villus, a shallower crypt and a disorganized structure. By contrast, the treatment with AFP moderated the damage to the intestinal mucosa caused by Cy, and was associated with a neat and compact villus. As shown in Figure 5A, the villus length of the AFP group was markedly increased compared to that in the MC group (*p* < 0.01). A longer villus could enhance the absorption of nutrients and the resistance ability to bacteria via contacting with intestinal epithelial cells. As Figure 5C indicates, the villus length/crypt ratio was enhanced significantly in the AFP groups compared to the MC group (*p* < 0.01), and was even comparable to the NC group (*p* > 0.05).

### 3.8. Effect of AFP on the Goblet Cells and PAS-Positive Area

**Figure 6:**
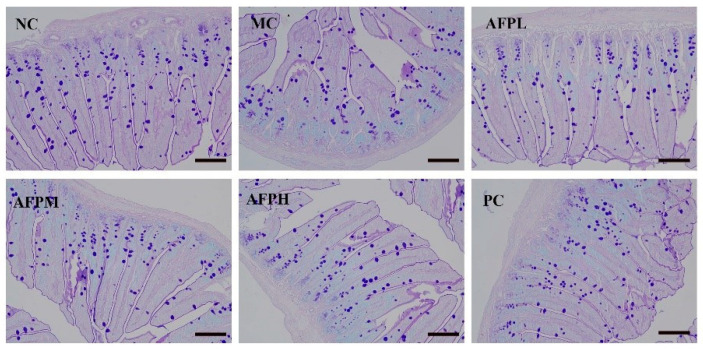
Representative images of the small intestine tissue AB-PAS staining sections (200×) among 6 mice, scale bar: 100 μm.

In this study, the state of goblet cells was evaluated using AB-PAS staining. Goblet cells were blue in the staining sections of the small intestine tissue. As exhibited in Figure 6, the numbers of goblet cells in the MC group were obviously inferior to that in the NC group, indicating that Cy caused severe damage to the intestinal mucous cells. In comparison with the MC group, the number of goblet cells was gradually restored after being treated with AFP, but was still lower than the NC group.

### 3.9. Effects of AFP on IgA and IgG Levels in the Serum

**Figure 7:**
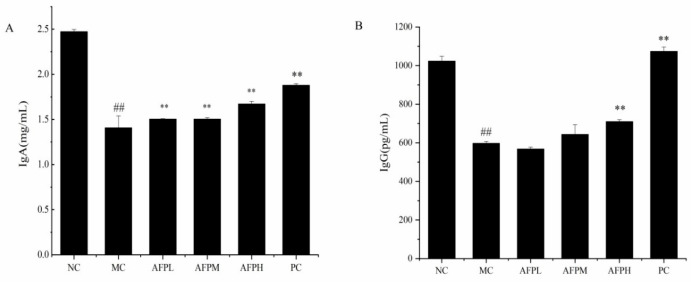
Effect of AFP on the serum IgA (**A**) and IgG (**B**) level in mice. Data are expressed as the mean ± SD (*n* = 6). ** *p* < 0.01 compared with MC; ^##^ *p* < 0.01 compared with NC.

As demonstrated in Figure 7, Cy significantly lessened the expression levels of IgA and IgG in serum in comparison to that of normal mice (*p* < 0.01). Inversely, the IgA and IgG content in the serum of mice tended to increase in a dose-dependent manner after being treated with AFP. AFP remarkably upregulated the expression of IgA in serum compared with that of the MC group (*p* < 0.01). The effect of the AFPH group was even similar to that of the PC group. Nevertheless, only the AFPH group was notably different from the MC group regarding the promotion of IgG’s expression (*p* < 0.01); meanwhile, the other two groups had no distinct effect on the secretion of IgG.

### 3.10. Effects of AFP on the Cytokine Levels in the Small Intestine

**Figure 8:**
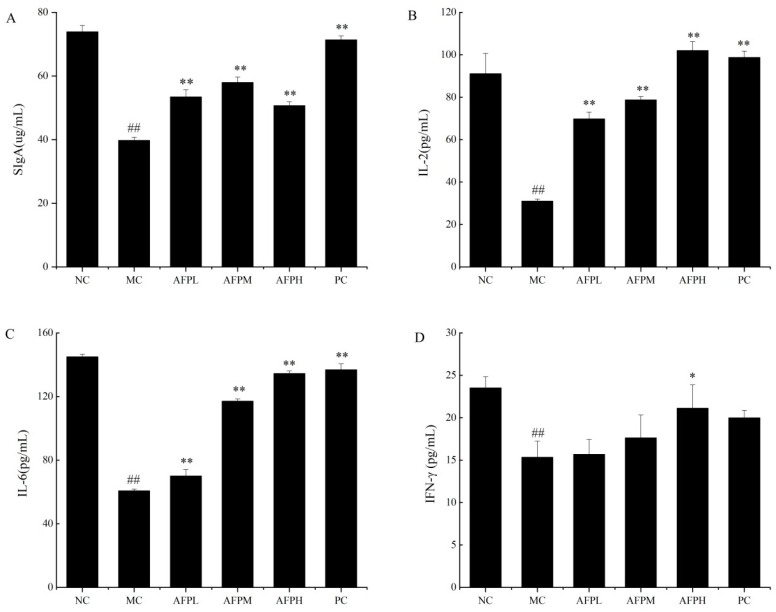
Effect of AFP polysaccharides on the small intestinal cytokine level in mice. (**A**) SIgA, (**B**) IL-2, (**C**) IL-6, (**D**) IFN-γ. Data are expressed as the mean ± SD (*n* = 6). * *p* < 0.05, ** *p* < 0.01 compared with MC; ^##^ *p* < 0.01 compared with NC.

To investigate the change in the small intestinal cytokines in immunosuppressed mice, the secretion of SIgA, IL-2, IL-6, and IFN-γ was determined via an ELISA kit, and the results are revealed in Figure 8. As expected, a sharp decline appeared in four small intestinal cytokines of the MC group, relative to the NC group (*p* < 0.01). This result suggests that the immune system was damaged by Cy in mice. As presented in Figure 8A, compared to the MC group, the AFP-treated groups secreted SIgA at a relatively higher level but did not show a dose-dependent increase (*p* < 0.01). The expression of IL-2 and IL-6 was remarkably enhanced in a dose-dependent manner in comparison with the MC group (*p* < 0.01), especially for the AFPH group (AFPH), the effect of which was equivalent to that of the PC group. In comparison with the MC group, the expression of IFN-γ in the AFPL and AFPM groups was increased to varying degrees without a significant difference; while the AFPH group showed a prominent increase (*p* < 0.05).

## 4. Discussion

As a conventional Chinese remedy, AF has been used for centuries to cure cardiovascular diseases, hypertension, fever, osteoporosis lung disease, and so on [18]. As reported, AFP, the important active component of the AF herb, possesses significant antitumor and antidiabetic activities. Much of the literature on this component focuses on the partial bioactivity of AFP. The immunosuppressive activity of AFP following oral administration is less clear. In this study, the immunosuppressive mice model induced by Cy was used to evaluate the immunomodulatory effect of AFP.

Macrophages are important effector cells of nonspecific immune response and are widely distributed in organisms [35]. Studies have shown that polysaccharides can not only enhance the proliferation of macrophages, but can also activate macrophages to release cytokines to resist or kill pathogens, thereby improving host immunity [36]. In our study, a dose-dependent enhancement of cell proliferation, IL-6, and TNF-α was observed in RAW 264.7 cells treated with 100 to 400 μg/mL doses of AFP. AFP has a significant immunomodulatory effect on macrophages.

Studies have shown that the immunomodulatory effects of polysaccharides on the body are mediated via different intracellular signaling pathways [37]. Therefore, we further explored the potential mechanisms by which AFP regulates the immune activity of the RAW 264.7 NF-κB transcription factor, which has been suggested to be directly associated with the expression of apoptosis, senescence, immunity and inflammation-related genes; it is also an essential signaling pathway that plays a crucial role in the immune system, of which, NF-κB p65 is the most important subunit and is involved in the expression and regulation of many genes as an indicator of activated cells [38]. Therefore, we analyzed the phosphorylation levels of NF-κB p65 in RAW 264.7 cells. In our study, the significantly enhanced phosphorylation expression levels of NF-κB p65 indicted the good activation of the NF- κB signaling pathway after AFP treatment. With an increase in the concentration, the best performance was obtained at the dosage of 400 μg/mL of AFP, which is 1.23-fold higher than the positive control group and 1.78-fold higher than the control group. These results suggest that NF-κB is a key signaling pathway via which AFP can exert immune activity in RAW 264.7 cells, and may account for the AFP-induced cytokine production in RAW 264.7 cells.

Body weight and the thymus index are basic physiological conditions of mice. The loss of body weight indicated that Cy caused a certain degree of damage to the mice, which is in accordance with the previous literature [39]. In addition, the decrease in the thymus index was a typical feature of immunosuppressed mice, on account of the weight of the immune organ correlating with the number of immune cells [40]. After the administration of AFP, the weight and the thymus index were enhanced, which demonstrated that polysaccharides can slow down the damage caused by Cy and restore the immune response.

A complete intestinal morphology is a prerequisite for the normal functioning of the mucosal barrier. The inner wall of the gastrointestinal tract has a layer of mucus that is produced by goblet cells, thus preventing pathogens from invading the mucosa and causing intestinal inflammation [41]. The current study found that the number of goblet cells in the MC group was clearly decreased compared to the NC group, indicating that Cy caused severe damage to intestinal mucous cells. However, the introduction of AFP helps to rebuild the secretion of goblet cells, but there is always room to improve compared with the normal group. Beneath the mucus layer, epithelial cells form another line of defense in the gut, preventing the invasion of pathogenic microorganisms [42]. Intestinal villus is a finger-like small protrusion of the epithelium cell protruding into the intestinal lumen. The length of the intestinal villus is positively correlated with the number of epithelial cells. In this study, the villus length was obviously shorter after treated with Cy, suggesting a decrease in the number of epithelial cells. According to the literature, as the villus becomes shorter, the number of epithelial cells decreases, resulting in the reduced digestion and uptake of nutrients [43]. The base of each villus is surrounded by crypts. The crypt depth reflects the regeneration rate of intestinal epithelial cells [44]. The researchers found that Moringa oleifera polysaccharides restored the villus height and crypt depth in a Cy-induced mice model of intestinal injury [45]. Consistent with previous studies, AFP promoted the regular and compact arrangement of the small intestinal villus, improved the integrity of the physical barrier of the small intestine and enhanced the villus length and the villus length/crypt ratio. Thus, we could confirm that AFP possesses pronounced immunomodulatory activities, and plays an essential role in enhancing the intestinal morphology of the immunological system in vivo.

Cy inhibited the generation of the antibody by mediating B cells. Levels of IgA and IgG were significantly decreased in immunosuppressed mice. However, both the IgA and IgG content in the serum of mice tended to increase in a dose-dependent manner after being treated with AFP. AFP remarkably upregulated the expression of IgA and IgG in serum compared with that of the MC group (*p* < 0.01), which is consistent with the results of a previous study [46]. Once the body was attacked by disease, the IgA and IgG levels became abnormal [47]. An increase in IgA and IgG helps the body to remove harmful antigens. On the basis of the above-mentioned results, it could be concluded that AFP regulates the immune activity of mice from the perspective of humoral immunity.

As the major class of antibody present on intestinal mucosal surfaces [48], SIgA provides an invaluable barrier that limits the access of intestinal antigens to the intestinal mucosa, controls the intestinal microbiota and attenuates pro-inflammatory immune responses. Similar to previous research [49], compared to the MC group, AFP-treated groups displayed a relatively higher secretion of SIgA, but no dose-related increase (*p* < 0.01). The results indicated that AFP could be used as a protective agent in order to alleviate Cy-induced intestinal damage and immune suppression in mice. To further investigate the regulatory effect of AFP on the intestinal mucosal barrier, the secretion of small intestinal cytokine in mice was measured. IL-2 mediates the activation of CD4^+^ and CD8^+^ T cells, induces the differentiation of T helper cells and facilitates the proliferation of immunoglobin synthesis by activated B cells [50]; while IL-6 plays a role in activating B-cells and plasma cells, involves in the differentiation into plasma cells, and produces IgG [51]. Our data highlighted that the levels of IL-2 and IL-6 were remarkably heightened compared to the MC group (*p* < 0.01), especially for the AFPH group, the effect of which was equivalent to that of the PC group. IFN-γ is mainly produced by NK/ILC1 cells and T cells, and plays an important role in the immune response to bacterial infections [52]. The results of this study revealed that, compared to the MC group, the expression of IFN-γ in the AFPL and AFPM groups was increased to varying degrees without a significant difference; this was different from AFPH group, which exhibited a prominent increase. These results indicated that AFP has a good alleviating effect on mice immunosuppressed by Cy.

## 5. Conclusions

In summary, AFP was extracted from *Anoectochilus formosanus* with a homogeneous *M_w_* distribution of 16.41 kDa using a water extraction and alcohol precipitation method. To the best of our knowledge, this was the first in-depth study of AFP’s immunoregulatory activity and its mechanism in immunosuppressed mice. This study confirmed that AFP has an ability to enhance the secretion of TNF-α and IL-6 in RAW 264.7 macrophages, as well as the production of cytokines (IgA, IgG, SIgA, IL-2, IL-6 and IFN-γ) in immunosuppressed mice. The western blot analysis showed that NF-κB signaling pathways were involved in macrophage activation induced by AFP. Moreover, AFP could accelerate the recovery of the thymus index and body weight. Additionally, the repair effects of AFP on the intestinal morphology of Cy-induced immunosuppressed mice demonstrated the ability of AFP to help the mice mitigate the immunotoxicity caused by Cy. Our investigations suggested that AFP might have immunomodulatory effects in vivo and in vitro.

These preliminary investigations will lay the theoretical groundwork for the subsequent study of functional food based on *Anoectochilus formosanus*. However, the interaction between the structure and immune activity of AFP needs further study.

## Figures and Tables

**Table 1:** Change in body weight of Cy-exposed mice.

Group	Initial Weight (g)	Weight on the Fourth Day (g)	Final Weight (g)	Weight Gain (g)
NC	18.14 ± 1.31	18.02 ± 0.95 *	18.59 ± 1.00	0.55 ± 0.69
MC	19.28 ± 1.07	17.20 ± 1.02 ^#^	18.56 ± 0.73	−0.72 ± 0.54
AFPL	18.63 ± 1.16	16.63 ± 1.15 ^#^	18.25 ± 0.69	−0.04 ± 0.84
AFPM	18.44 ± 1.02	16.60 ± 1.40 ^#^	17.90 ± 0.82	−0.17 ± 0.61
AFPH	18.63 ± 0.81	16.57 ± 0.93 ^#^	17.81 ± 0.88	−0.48 ± 0.32
PC	19.29 ± 1.12	17.26 ± 0.87 ^#^	18.69 ± 1.04	−0.38 ± 0.52

The values are presented as mean ± SD, *n* = 10. * *p* < 0.05, compared with MC. ^#^ *p* < 0.05, compared with initial weight.

## Data Availability

Data is contained within the article.
